# COVID-19 phobia in a boy with undiagnosed autism spectrum disorder: A case report

**DOI:** 10.1097/MD.0000000000026233

**Published:** 2021-06-04

**Authors:** Shoko Sakamoto, Dai Miyawaki, Ayako Goto, Yuji Harima, Daisuke Tokuhara, Koki Inoue

**Affiliations:** a Department of Neuropsychiatry; b Department of Pediatrics, Osaka City University Graduate School of Medicine, Asahi-machi, Abeno-ku, Osaka, Japan.

**Keywords:** anxiety disorders, autism, avoidant restrictive food intake disorder, case report, coronavirus disease 2019

## Abstract

**Rationale:**

Coronavirus disease 2019 (COVID-19) is affecting mental health profoundly. Previous studies have reported pandemic-related anxiety. Anxiety disorder and autism spectrum disorder (ASD) are common comorbidities. However, there has been no report of any patient with undiagnosed ASD who developed anxiety disorders caused by the COVID-19 pandemic. In this case report, we describe an 8-year-old Japanese boy with undiagnosed ASD who developed COVID-19 phobia, resulting in avoidant restrictive food intake disorder (ARFID).

**Patient concerns:**

As COVID-19 was highly publicized in the mass media and the risk of droplet infection was emphasized upon, the patient began to fear viral contamination from food, culminating in a refusal to eat or even swallow his saliva. He was admitted to a pediatric medical center in Osaka with life-threatening dehydration and was then referred to our child psychiatry department.

**Diagnosis:**

We diagnosed the patient with COVID-19 phobia resulting in ARFID. We identified ASD traits from his present social communication skills and developmental history.

**Interventions:**

We provided psychoeducation of ASD for the parents and administered supportive psychotherapy.

**Outcomes:**

Shortly after our intervention to relieve his ASD-related anxiety, his dysphagia improved.

**Lessons:**

Our findings suggest that children with undiagnosed ASD may develop COVID-19 phobia. In these cases, intervention for ASD may be more appropriate than starting treatment for anxiety disorders as the first-line option. COVID-19 is the biggest pandemic in the recent past, and more undiagnosed ASD patients who develop COVID-19 phobia may seek treatment. Clinicians should consider the underlying ASD in these patients and assess their developmental history and present social communication skills.

## 1 Introduction

It is already evident that the psychological effects of the coronavirus disease 2019 (COVID-19) pandemic are pervasive and could affect mental health by exacerbating anxiety symptoms at present and in the future.^[1,2]^ At the outbreak of the severe acute respiratory syndrome^[3]^ and H1N1 influenza,^[4]^ pandemic illness-related anxiety was reported. Anxiety is common in individuals with autism spectrum disorder (ASD).^[5]^ However, there has been no report of any patient with undiagnosed ASD who developed anxiety disorders caused by the pandemic. In this case report, we describe COVID-19 phobia resulting in avoidant restrictive food intake disorder (ARFID) in an 8-year-old Japanese boy with undiagnosed ASD. Our treatment strategy, targeting the ASD characteristics and the overlooked distress related to social communication impairment, improved his dysphagia.

## 2 Case report

The patient was an 8-year-old Japanese boy with no history of eating disorders. As COVID-19 was highly publicized in the mass media 2 months after the first outbreak, he feared its infection so much that he refused to go anywhere, even to school. He washed his hands and rinsed his mouth excessively. He lost his temper when his family did not take sufficient infection control measures at home, such as wearing a mask or talking gently without spitting out. After learning about the risk of its droplet infection, he began to fear viral contamination from food, culminating in a refusal to eat for 3 weeks. He was unable to even swallow his own saliva. Due to life-threatening dehydration, he was admitted to a pediatric medical center in Osaka following a week of outpatient pediatric treatment with no improvement and was referred to our child psychiatry department for psychiatric treatment.

After admission, he received physical treatment, such as intravenous feeding by pediatricians without any fear of getting infected, in parallel with intervention by a psychiatrist. He kept smiling at the medical staff even when he was troubled but did not answer any questions, indicating atypical social communication. To evaluate his developmental history, we comprehensively interviewed the patient and his mother. At the age of 2, he had little interest in other children or joint attention. At the age of 3, he passively attended nursery school but tended to engage in stereotyped play and hated to be disturbed by others. He wanted to avoid novel situations and rarely asked his teachers or classmates for help despite facing difficulties. He also exhibited hypersensitivity to sound and smell. Nevertheless, his development was assessed as normal in medical checkups at 18 months and 3 years of age because he had no obvious delayed speech. His intelligence and language abilities were normal (Wechsler Intelligence Scale for Children IV: Full-Scale IQ = 95). We used the Pervasive Developmental Disorders Autism Society Japan Rating Scale (PARS),^[6]^ a standardized semi-structured interview useful for children and adults, and the Japanese version of the Schedule for Affective Disorders and Schizophrenia for School-Age Children – Present and Lifetime Version ^[7]^ for assessment. He has been exceeding the cutoff score for ASD on PARS since early childhood and to the present. We confirmed that he had persistent deficits in social communication, social interaction, and relationship development. He also had restricted and repetitive interests, and hyperreactivity to sensory inputs. Subsequently, we diagnosed ASD based on the *Diagnostic and Statistical Manual of Mental Disorders*, Fifth Edition (*DSM-5*) criteria. The total clinical evaluation showed that he had COVID-19 phobia, resulting in ARFID. Thus, we suggest that overlooked social difficulties related to undiagnosed ASD serve as a predisposing factor for the development of COVID-19 phobia resulting in ARFID.

The treatment approach that we employed focused on the patient's ASD traits. We told his parents the preference for specific and simple language use in children with ASD and that verbal threat information about COVID-19 (i.e., the number of infected people or deaths) from the media could promote his fear because of his literal interpretation, and that blaming him for refusing to eat was not effective. Further, we told the patient some information regarding the treatment setting, such as an average weekly schedule, available items, and when he can use a playing room, trying to make him feel comfortable. Medical staff showed him the menu before each meal to decrease his anxiety and to ensure the safety of the food with no relation to COVID-19 infection. As his fear faded, his dysphagia improved significantly, and he was able to orally consume more than half of his food. We discharged him in 2 weeks and continued his treatment in our outpatient clinic. We administered supportive psychotherapy to him and educated his parents and teachers about ASD and its supportive methods. We refrained from open-end questions not to make him confused and gave special care, such as sound, seating formation, regular appointment times. The food intake and his body weight returned to pre-onset levels within 2 months. Currently, the patient has become more adaptive at school through an individual education program and has been in complete remission of COVID-19 phobia for more than a year despite the ongoing pandemic. Written informed consent and assent were obtained from the patient and parents according to the IRB regulation.

## 3 Discussion

To the best of our knowledge, this is the first report of COVID-19 phobia in a child with undiagnosed ASD and provides two important insights. First, children with undiagnosed ASD may develop COVID-19 phobia following the recent pandemic. Second, in diagnosed cases, intervention for ASD, such as sharing information with parents and teachers to explain what autism is and how it is likely to affect the child's development and function and to make an individual education plan may be more appropriate than starting treatment for anxiety disorders as the first-line option.

Children with ASD suffer from social difficulties related to the traits,^[8]^ and we suggest that the overlooked distress in undiagnosed cases can be a predisposing factor for the development of anxiety disorders. Children with ASD often show anxiety, which Boulter et al. (2014) pointed out to be associated with intolerance to uncertainty.^[9]^ Children with ASD interpret all ambiguous information as threatening, contributing to significant somatic stress responses in the face of novel or uncertain situations.^[9]^ It is highly possible that children with ASD are more vulnerable to verbal threat information from the media because they interpret it literally. In our case, although nobody in his vicinity had been infected with COVID-19, he has been excessively anxious, possibly worsened by overwork in the media. In undiagnosed cases, children do not have any support for their ASD traits, and anxiety can exacerbate.^[8]^ The effect of the COVID-19 pandemic on mental health concerns, including anxiety symptoms, has been reported,^[1]^ which can continue to increase in such an unprecedented condition. Even during the outbreak of the severe acute respiratory syndrome^[3]^ and H1N1 influenza,^[4]^ pandemic-related anxiety was reported to be associated with high publicity in the mass media.

Many children with ASD remain undiagnosed, as early symptoms of ASD can easily be overlooked.^[10,11]^ Among ASD patients, there are significant differences in phenotypic expressions, associated with high rates of comorbidity with other psychiatric disorders.^[12]^ Psychiatric comorbidities can also obscure an underlying ASD and delay its diagnosis, although in some cases, ASD can be diagnosed before the age of 3.^[11]^ We, therefore, expect that more undiagnosed ASD patients developing anxiety disorders caused by the pandemic may seek treatment.

Our findings suggest that intervention for ASD may be more appropriate than starting treatment for anxiety disorders as the first-line option in children with ASD who developed COVID-19 phobia caused by the pandemic. We simply helped the parents and teachers understand the ASD characteristics, such as literal interpretation and hypersensitivity. As we understood he might likely feel anxious about uncertainty, we gave him clear information with visual support in advance regarding the treatment setting where could be over-stimulating for children with ASD. We allowed him to play by himself, not in a group, for his personal space. After our treatment, his anxiety was relieved, and dysphagia improved. Spain et al reported that for individuals with an ASD and an anxiety disorder but no intellectual disability, cognitive behavioral therapy modified by the direct instruction of social skills, increased family involvement, visual supports, individualized reinforcers, embedded perseverative interests in sessions, and reduced emphasis on abstract concepts and visualization have been supported by research.^[13]^ In cases of ASD with anorexia nervosa, which is a relatively common comorbidity, the presence of underlying ASD traits, such as rigidity and low introspection, effectively maintains AN.^[14]^ Thus, the patients require adapted or targeted treatment programs.^[15]^ A previous study reported that cognitive remediation therapy to address problems with cognitive style and meta-cognition by stimulating the neural connections involved in cognitive processing through cognitive tasks, reflection, and behavioral experiments, was efficient for treating patients with ASD and anorexia nervosa.^[16]^ Their findings support our contention that intervention for ASD may be more appropriate than starting treatment for anxiety disorders as the first-line option in children with ASD who developed COVID-19 phobia caused by the pandemic. Our strength is that we diagnosed ASD based on the *DSM-5* criteria through the standardized semi-structured interview. However, the therapeutic potential in treating these cases needs to be further examined in future studies.

## 4 Conclusion

Children with undiagnosed ASD may develop COVID-19 phobia due to the recent pandemic. Several patients developing ARFID should be considered to have undiagnosed ASD. In these cases, intervention for ASD, such as sharing information with parents and teachers to explain what autism is and how it is likely to affect the child's development and function, and making individual education plans may be more appropriate than starting treatment for anxiety disorders as the first-line option. COVID-19 is the biggest pandemic in the recent past, and more undiagnosed ASD patients who develop COVID-19 phobia may seek treatment. Clinicians should consider underlying ASD in the patients and assess their developmental history, such as joint attention, as well as their present social communication skills. Further studies are required to clarify the prevalence of ASD in patients with COVID-19 phobia, and a large controlled study is required to fully evaluate the effectiveness of our clinical strategies.

## Acknowledgments

The authors thank the patient and his parents for their agreement to publish this study.

## Author contributions

SS produced the initial draft and interpreted the case findings. DM, AG, YH, DT, and KI critically revised the draft. All authors read and approved the final manuscript.

**Supervision:** Koki Inoue.

**Writing – original draft:** Shoko Sakamoto.

**Writing – review & editing:** Dai Miyawaki, Ayako Goto, Yuji Harima, Daisuke Tokuhara.
